# SKN-1 Is a Negative Regulator of DAF-16 and Somatic Stress Resistance in *Caenorhabditis elegans*

**DOI:** 10.1534/g3.120.401203

**Published:** 2020-03-11

**Authors:** Jianhui Deng, Yuxi Dai, Haiqing Tang, Shanshan Pang

**Affiliations:** School of Life Sciences, Chongqing University, Chongqing, 401331, China

**Keywords:** SKN-1/Nrf2, DAF-16, stress, aging, lipid metabolism

## Abstract

The transcription factor SKN-1, the *C. elegans* ortholog of mammalian Nrf protein, is a well-known longevity factor, and its activation is observed in several long-lived models. SKN-1 also plays essential roles in xenobiotic and oxidative stress responses. Here, we report deleterious functions of SKN-1 in somatic stress resistance that may impair lifespan. Constitutive SKN-1 activation impairs animal resistance to several stresses, including heat, ER stress and mitochondrial stress, which result from the suppression of DAF-16, another master regulator of longevity. SKN-1 activation abrogates DAF-16 nuclear import and downregulates DAF-16 target genes under stress conditions, while SKN-1 inhibition promotes the expression of DAF-16 targets, even in long-lived mutants. Further, SKN-1 activation induces the expression of vitellogenin proteins, which are required for SKN-1-mediated suppression of DAF-16 and stress resistance. Together, these findings identify detrimental roles for SKN-1 activation in animal health, and more importantly, inspire the rethinking of the complex roles for SKN-1 in aging regulation.

Transcription factors play crucial roles in the regulation of the aging process. Diverse environmental cues and endogenous genetic alterations converge on limited core transcription factors to promote longevity. SKN-1, the *C. elegans* ortholog of mammalian Nrf proteins, is one of these longevity transcription factors. The activation of SKN-1 is observed in several long-lived mutants and is required for lifespan extension (Bishop and Guarente 2007; Tullet *et al.* 2008; Steinbaugh *et al.* 2015). SKN-1 activation promotes *C. elegans* resistance to several aging-related stresses, including xenobiotic and proteostatic stresses (An and Blackwell 2003; Li *et al.* 2011; Lehrbach and Ruvkun 2019). Moreover, the repression of Nrf2 contributes to premature aging in mammals, while the activation of Nrf2 reverses several aging defects observed in Hutchinson-Gilford progeria syndrome (Kubben *et al.* 2016). These findings together highlight the conserved anti-aging effects of SKN-1/Nrf in animals.

However, recent works in *C. elegans* suggest that the constitutive activation of SKN-1 has pleiotropic effects on animal health. *C. elegans* harboring *skn-1* gain-of-function (*skn-1* gof) alleles unexpectedly exhibit age-dependent somatic depletion of fat (Asdf) (Lynn *et al.* 2015), which compromises animal health in later life. Additionally, the *skn-1* gof animals even exhibit a slightly shortened lifespan (Paek *et al.* 2012), implying the possible harmful effects of SKN-1 on animal aging. However it is unknown whether SKN-1 activation has detrimental effects on aging-related phenotypes that are independent of Asdf. In this study, we explored this question and reported the suppressive effects of SKN-1 on animal stress responses that involve the transcription factor DAF-16, supporting dual roles for SKN-1 in aging regulation.

## Materials and Methods

### C. elegans strains and maintenance

*C. elegans* were cultured on standard nematode growth medium (NGM) seeded with *E. coli* OP50-1 (Brenner 1974). The following strains were provided by Caenorhabditis Genetics Center: wild type N2 Bristol, SPC207[*skn-1*(*lax120*)], SPC227[*skn-1**(**lax188**)*], CF1553[*sod-3p*::*gfp*], TJ356[*daf-16p*::*daf-16a/b*::*gfp*], CF1038 [*daf-16*(*mu86*)], VC1772[*skn-1*(*ok2315*)/nT1 (qIs51)], DR1572 [*daf-2*(*e1368*)], RT130[*vit-2*::*gfp*], RB2365 [*vit-2*(*ok3211*)], RB2202 [*vit-4*(*ok2982*)], RB2382[*vit-5*(*ok3239*)], CB4037[*glp-1*(*e2141*)], CL2166[*gst-4p*::*gfp*], LD1[*skn-1b/c*::*gfp*]. The strain SSP171[*mtl-1*::*gfp*] was generated by cloning the full length of *mtl-1* genomic DNA into pPD95.79 plasmid that was injected into gonad by standard techniques. Extrachromosomal arrays were integrated into genome by UV irradiation. Double mutants were generated by standard genetic techniques.

### Stress resistance assays

For heat shock resistance assay, day 1 adult worms were cultured at 35° for survival analysis unless otherwise indicated. For TBHP (Sigma) resistance, day 1 adult worms were transferred to NGM plates supplemented with 10mM TBHP and incubated at 20° for survival analysis. For DTT resistance, day 1 adult worms were transferred to NGM plates supplemented with 7.5mM DTT and incubated at 20° for survival analysis. For MA (sigma) resistance, day 1 adult worms were transferred to NGM plates supplemented with 34mM MA and incubated at 20° for survival analysis.

### Fluorescent microscopy

For fluorescent analysis, VIT-2::GFP worms were examined at 20° and other GFP worms were exposed to 31° heat stress for 8-12 hr and then collected for imaging. Microscopic imaging was performed as previously described (Tang and Pang 2016). To analyze the expression of *sod-3p*::GFP, MTL-1::GFP or *gst-4p*::GFP expression, worms were paralyzed with 1mM levamisole and fluorescent microscopic images were taken after mounted on slides. To study the DAF-16 or SKN-1 nuclear localization, DAF-16::GFP or SKN-1::GFP worms were mounted on slides. The levels of GFP nuclear localization were scored. Briefly, no nuclear GFP, GFP signal in the nucleus of anterior or posterior intestine cells and nuclear GFP in all intestinal cells are categorized as low, medium and high expression, respectively.

### RNA interference treatment

HT115 bacteria containing specific double-stranded RNA (dsRNA)-expression plasmids were seeded onto NGM plates containing 5mM isopropyl b-D-1-thiogalactopyranoside (IPTG). RNAi was induced at 25° for 24 hr. L1 worms were added to those plates to knockdown indicated genes.

### qRT-PCR

qRT-PCR was performed as previously described (Tang and Pang 2016; Zhou *et al.* 2019). Briefly, day 1 adult WT and the *skn-1* gof mutant worms were collected, washed in M9 buffer and then homogenized in Trizol reagent (Life Technologies). RNA was extracted according to manufacturer’s protocol. DNA contamination was digested with DNase I (Thermo Fisher Scientific) and subsequently RNA was reverse-transcribed to cDNA by using the RevertAid First Strand cDNA synthesis Kit (Thermo Fisher Scientific). Quantitative PCR was performed using SYBR Green (Bio-Rad). The expression of *snb-1* was used to normalize samples.

### Fatty acid quantification

Fatty acid contents were measured as previously described (Brock *et al.* 2006) with some modifications. 500-1,000 age-synchronized day 1 adult worms were washed off plates and washed three times with water. Worm pellets were resuspended with 1.2 mL of 2.5% H_2_SO_4_ in methanol and incubated at 80° for 1 hr. Then, 1 ml supernatant was mixed with 1.2 ml hexane and 1.8 ml water to extract fatty acid methyl esters for GC-MS analysis. The Supelco 37 Component FAME Mix (Sigma) was used to determine the retention time. The *Shimadzu GCMS-TQ8040* Gas Chromatograph Mass Spectrometer equipped with SH-Rxi-5sil MS column was used.

### Oil red O staining

Oil red O staining were performed as previously described (Wählby *et al.* 2014). Day 1 adult worms were collected and fixed in 60% isopropanol for 5 min. Worms were washed with M9 buffer and collected for staining with freshly prepared Oil Red O working solution (40% water with 60% stock solution of 0.5% Oil red O in isopropanol). After overnight staining, worms were washed twice with M9 buffer, mounted on slides and imaged under a bright-field illumination.

### Quantification and statistical analysis

Data were presented as mean ± SEM. Survival data were analyzed by using log-rank (Mantel-Cox) tests. The levels of fluorescent micrographs were analyzed by using Chi-square and Fisher’s exact tests. The qPCR and GC-MS data were analyzed by using paired student *t*-tests. *P* < 0.05 was considered as significant.

### Data availability

Strains are available upon request. The authors affirm that all data necessary for confirming the conclusions of the article are present within the article, figures, and tables. Supplemental Figure S1, S2, S3 and Table S1, S2 have been uploaded to figshare at https://doi.org/10.25387/g3.11919495.

## Results and Discussion

### SKN-1 activation compromises stress resistance and suppresses DAF-16

**Figure 1:**
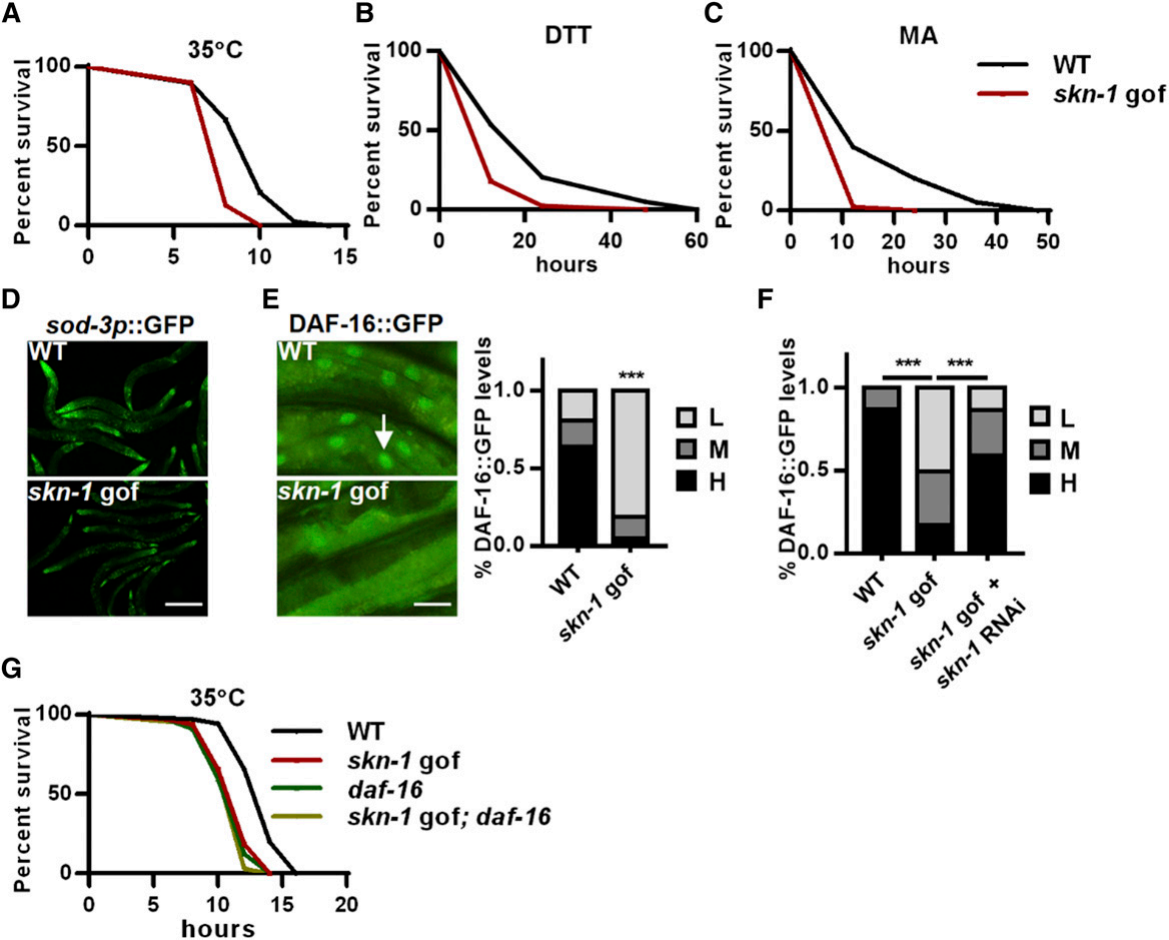
SKN-1 activation suppresses DAF-16 and impairs stress resistance. (A-C) The *skn-1* gof mutation *skn-1**(**lax120**)* impaired animal resistance to the heat stress 35°C (A), dithiothreitol (DTT)-induced ER stress (B) and malonic acid (MA)-induced mitochondrial stress (C). (D-E) Effects of the *skn-1* gof mutation on the expression of *sod-3p*::GFP (D), and the nuclear accumulation of DAF-16::GFP (E) under heat stress. Left panel: representative images; right panel: quantification data. Arrow indicates nuclear GFP. The nuclear occupancy of DAF-16::GFP were scored as low (L), medium (M) and high (H). (F) *skn-1* RNAi abrogated the suppressive effects of the *skn-1* gof mutation on DAF-16::GFP nuclear accumulation. (G) Effects of the *skn-1* gof mutation and the *daf-16* null mutation were not additive on heat stress resistance. *** *P* < 0.001. Scale bar = 100μm for D. Scale bar = 20μm for E.

Improved stress resistance is believed to be beneficial for longevity. As such, we explored the effects of SKN-1 activation on multiple stress resistance in early adulthood (day 1 adult) *skn-1* gof mutants. The *skn-1* gof animals exhibited improved resistance to oxidative stress (Fig S1A), which was consistent with the dominant role of SKN-1 in xenobiotic response (An and Blackwell 2003; Oliveira *et al.* 2009; Nhan *et al.* 2019). Strikingly, SKN-1 activation led to the dramatic inhibition of animal resistance to several other stresses, including heat, endoplasmic reticulum (ER) stress and mitochondrial stress (Figure 1A-1C), all of which are crucial for aging regulation. The suppressive effects of SKN-1 activation on stress resistance were confirmed by using an independent *skn-1* gof allele (Fig S1B and S1C) (Paek *et al.* 2012) and by RNAi of *wdr-23* (Fig S1D and S1E), a principle negative regulator of *skn-1* (Choe *et al.* 2009). These findings support the view that the constitutive activation of SKN-1, a classic longevity factor, has pleiotropic outcomes for animal health.

### SKN-1 activation suppresses DAF-16

In the following study, we chose the heat stress response for further mechanistic study. In *C. elegans*, stress responses are controlled by limited core transcription factors, such as DAF-16, the activation of which promotes multiple stress responses and longevity (Kenyon 2010). We therefore examined the effect of SKN-1 on DAF-16 activation upon heat stress and first measured the expression of two well-known DAF-16 reporters, *sod-3p*::GFP (Libina *et al.* 2003) and *mtl-1*::GFP. Remarkably, SKN-1 activation caused strong inhibition of DAF-16 reporters (Figure 1D and S1F), implying that SKN-1 may be a negative regulator of DAF-16. Moreover, the mRNA expression of several DAF-16-induced genes (Murphy *et al.* 2003) was suppressed by the *skn-1* gof mutation (Fig S1G), while the mRNA levels of *daf-16* were not affected (Fig S1H), suggesting that SKN-1 does not directly regulate *daf-16* transcription. We also compared the published genome-wide expression data (Oliveira *et al.* 2009; Tepper *et al.* 2013) and found that a significant proportion of the SKN-1-upregulated genes (66/233, 28.3%, *P* < 0.001) were the genes that are downregulated when DAF-16 is activated, confirming that a number of genes are oppositely regulated by SKN-1 and DAF-16. As a transcription factor, DAF-16 translocates to the nucleus upon activation. We found that the *skn-1* gof mutation significantly suppressed DAF-16 nuclear accumulation under heat stress (Figure 1E), while *skn-1* RNAi largely abolished this suppression (Figure 1F). Moreover, both the *skn-1* gof and *daf-16* loss-of-function mutations compromised the heat stress resistance in *C. elegans*, and their effects were not additive (Figure 1G). These data collectively suggest that the activation of SKN-1 can suppress the stress response regulator DAF-16.

### SKN-1 is suppressed by heat stress

We next examined SKN-1 regulation in response to heat stress. Intriguingly, heat exposure suppressed the expression of *gst-4p*::GFP (Fig S2A), a well-established SKN-1 reporter (Leiers *et al.* 2003), while the *skn-1* gof mutation abrogated the suppression (Fig S2B). Germline-deficient worms exhibited strong nuclear signals of intestinal SKN-1::GFP, facilitating the observation of SKN-1 nuclear occupancy. We also observed that SKN-1 nuclear accumulation in germline-deficient worms was abolished by heat stress (Fig S2C), indicating the silencing of SKN-1 during heat stress. SKN-1 expression can be activated by DAF-16 overexpression (Tullet *et al.* 2017). However, the suppression of SKN-1 did not require DAF-16, as *gst-4p*::GFP expression was inhibited in *daf-16* mutants when exposed to heat stress (Fig S2D). These data suggest that SKN-1 is suppressed during heat stress.

### SKN-1 inhibition activates DAF-16 and promotes heat stress resistance

**Figure 2:**
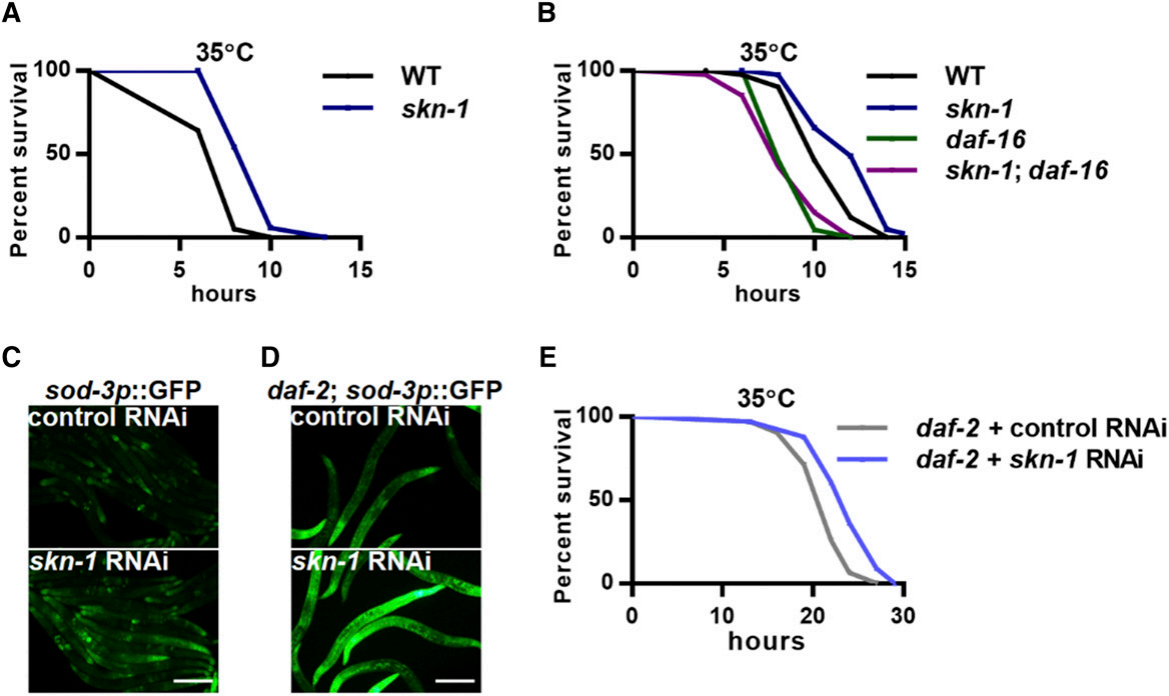
SKN-1 inhibition activates DAF-16. (A) Effects of the *skn-1* loss-of function mutation on heat stress resistance. (B) The *daf-16* null mutation abolished the enhancement of heat stress resistance in the *skn-1* loss-of-function mutants. (C) Effects of *skn-1* RNAi on the expression of *sod-3p*::GFP upon heat stress. (D-E) Effects of *skn-1* RNAi on the expression of *sod-3p*::GFP (D) and survival (E) of *daf-2* mutants under heat stress. Scale bar = 100μm.

We next asked whether the inhibition of SKN-1 could promote DAF-16 activity and heat stress resistance. As expected, SKN-1 inhibition, either through a loss-of-function mutation or a RNAi-mediated inhibition, significantly improved animal heat stress resistance (Figure 2A and S3A). More importantly, the enhancement of heat resistance required DAF-16, as the *skn-1* loss-of-function mutation failed to promote survival in *daf-16* mutants (Figure 2B). RNAi of *skn-1* also increased the expression of the DAF-16 reporters *sod-3p*::GFP and *mtl-1*::GFP (Figure 2C and S3B). Generally, SKN-1 and DAF-16 are activated simultaneously in long-lived mutants, including the insulin/IGF receptor mutant *daf-2* (Lin *et al.* 2001; Lee *et al.* 2001; Tullet *et al.* 2008) and the germline-deficient mutant *glp-1* (Lin *et al.* 2001; Steinbaugh *et al.* 2015). The reason for their coactivation is still unclear. We found that *skn-1* inhibition further increased the expression of *sod-3p*::GFP in both *daf-2* and *glp-1* mutants (Figure 2D and S3C) and further enhanced the heat stress resistance in these long-lived mutants (Figure 2E and S3D), suggesting a general suppression of DAF-16 by SKN-1. This may help to explain the coactivation of SKN-1 and DAF-16 in these long-lived mutants. SKN-1 activation alone may be deleterious, but when DAF-16 is concurrently activated, its negative effects on lifespan (suppression of DAF-16) are neutralized and its beneficial effects (promotion of xenobiotic response) become dominant.

### Unsaturated fatty acids mediate the suppressive effects of SKN-1 on DAF-16

**Figure 3:**
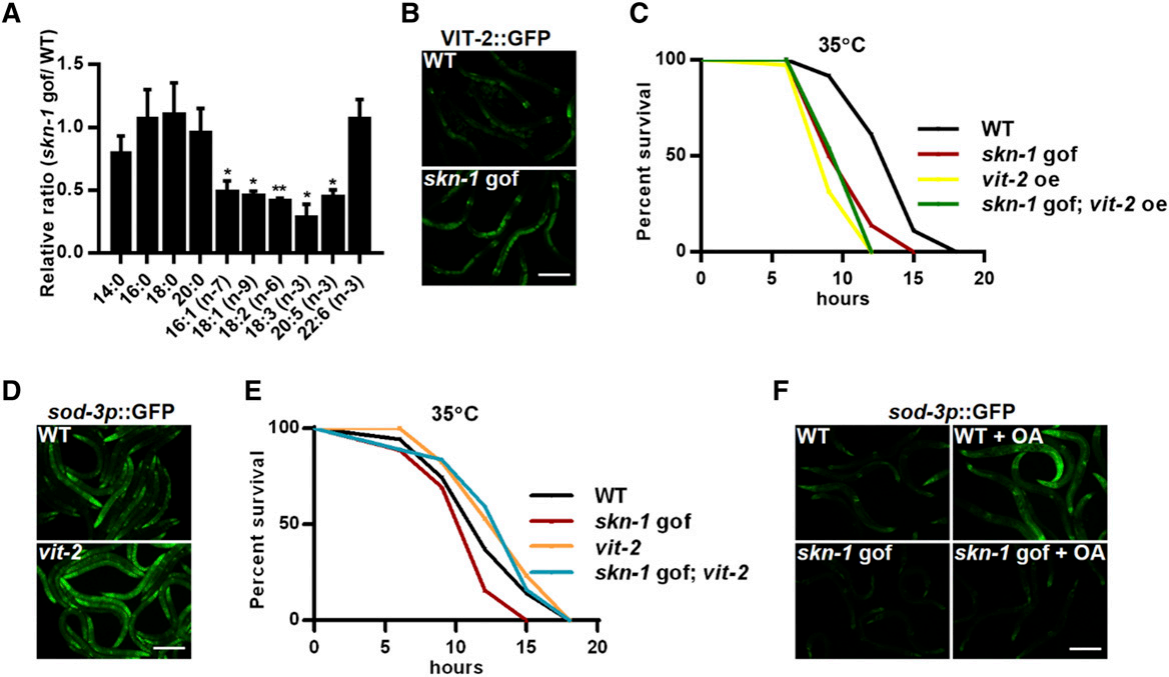
Lipid metabolism mediates the suppressive effects of SKN-1 on DAF-16. (A) Effects of the *skn-1* gof mutation on the contents of major fatty acid species. (B) Effects of the *skn-1* gof mutation on VIT-2::GFP expression. (C) *vit-2* overexpression (oe) impaired heat stress resistance, which was not additive with the *skn-1* gof mutation. (D) *vit-2* mutation increased the expression of *sod-3p*::GFP under heat stress. (E) *vit-2* mutation abolished the suppressive effects of the *skn-1* gof mutation in day 5 adult worms. (F) OA supplementation increased the expression of *sod-3p*::GFP in wild type but not in the *skn-1* gof mutant worms. * *P* < 0.05, ** *P* < 0.01. Scale bar = 100μm.

What is the possible mechanism for DAF-16 suppression in response to SKN-1 activation? SKN-1 is an important regulator of lipid metabolism (Paek *et al.* 2012; Pang *et al.* 2014; Steinbaugh *et al.* 2015; Nhan *et al.* 2019). The fat contents and distribution in the day 1 *skn-1* gof mutants are comparable to those in wild-type controls (Lynn *et al.* 2015). Our Oil Red O staining data confirmed that no Asdf phenotype occurred in the day 1 *skn-1* gof mutants (Fig S4A). Notably, GC-MS/MS analysis revealed reductions in many fatty acid species, mostly unsaturated ones (Figure 3A), indicating massive changes in lipid metabolism. The Asdf phenotype in *skn-1* gof mutants requires the yolk lipoprotein vitellogenins that transport the intestinal fat to the germline (Lynn *et al.* 2015). Additionally, a reduction in vitellogenins is reported to increase somatic fat stores, activate DAF-16 and extend lifespan (Seah *et al.* 2016). As such, we further tested whether vitellogenins also mediated the suppression of DAF-16 in early adulthood. The *C. elegans* genome encodes six vitellogenin genes (*vit-1** to **vit-6*). Using a *vit-2*::GFP strain, we found that SKN-1 activation increased the protein levels of VIT-2 (Figure 3B), and the overexpression of *vit-2* compromised heat stress response (Figure 3C), which phenocopied the *skn-1* gof mutation. Moreover, the effects of *skn-1* gof and *vit-2* overexpression were not additive (Figure 3C). Consistently, mutations of *vit* genes (*vit-2*, *vit-4* and *vit-5*) activated the DAF-16 reporter *sod-3p*::GFP (Figure 3D and S4B), and the *vit-2* mutation largely abolished the inhibitory effects of SKN-1 on heat stress resistance (Figure 3E). Therefore, the suppression of DAF-16 requires vitellogenins. Furthermore, since the *skn-1* gof mutation reduced the contents of unsaturated fatty acids (Figure 3A), we supplemented worms with oleic acid (OA), which is the first product of the unsaturated fatty acid biosynthetic pathway (Fig S4C) (Watts and Ristow 2017), and examined *sod-3p*::GFP expression. OA supplementation indeed increased the *sod-3p*::GFP activation (Figure 3F) in wild-type controls, while the effects were abolished by the *skn-1* gof mutation (Figure 3F); this might be caused by the constitutive activation of SKN-1 in *skn-1* gof mutants that reduced the exogenously supplemented OA. Together, these data are consistent with the idea that DAF-16 is regulated by OA (or its derivatives) and that the constitutive activation of SKN-1 results in the continual mobilization of OA, which likely accounts for DAF-16 inhibition and compromised stress resistance.

In summary, our study reports deleterious functions of SKN-1 in animal health through the interaction with DAF-16, supporting dual roles for SKN-1 in aging regulation. We propose that the lifespan extension effects of SKN-1 can only be manifested under specific conditions, such as in the context of DAF-16 activation, suggesting that different transcription factors must be coordinately regulated to ensure an optimal outcome for longevity, as in the insulin/IGF mutant and germline-deficient models.
